# A model-based estimation of critical torques reduces the experimental effort compared to conventional testing

**DOI:** 10.1007/s00421-020-04358-w

**Published:** 2020-04-10

**Authors:** Johannes L. Herold, Andreas Sommer

**Affiliations:** Interdisciplinary Center for Scientific Computing (IWR), Heidelberg University, Im Neuenheimer Feld 205, 69120 Heidelberg, Germany

**Keywords:** Critical torque, Elbow flexors, Isometric contractions, Optimum experimental design, Ordinary differential equation model

## Abstract

**Purpose:**

Critical torque (CT) is an important fatigue threshold in exercise physiology and can be used to analyze, predict, or optimize performance. The objective of this work is to reduce the experimental effort when estimating CTs for sustained and intermittent isometric contractions using a model-based approach.

**Materials and methods:**

We employ a phenomenological model of the time course of maximum voluntary isometric contraction (MVIC) torque and compute the highest sustainable torque output by solving an optimization problem. We then show that our results are consistent with the steady states obtained when simulating periodic maximum loading schemes. These simulations correspond to all-out tests, which are used to estimate CTs in practice. Based on these observations, the estimation of CTs can be formulated mathematically as a parameter estimation problem. To minimize the statistical uncertainty of the parameter estimates and consequently of the estimated CTs, we compute optimized testing sessions. This reduces the experimental effort even further.

**Results:**

We estimate CTs of the elbow flexors for sustained isometric contractions to be 28% of baseline MVIC torque and for intermittent isometric contractions consisting of a 3 s contraction followed by 2 s rest to be 41% of baseline MVIC torque. We show that a single optimized testing session is sufficient when using our approach.

**Conclusions:**

Our approach reduces the experimental effort considerably when estimating CTs for sustained and intermittent isometric contractions.

## Introduction

### Critical power and critical torque

**Fig. 1:**
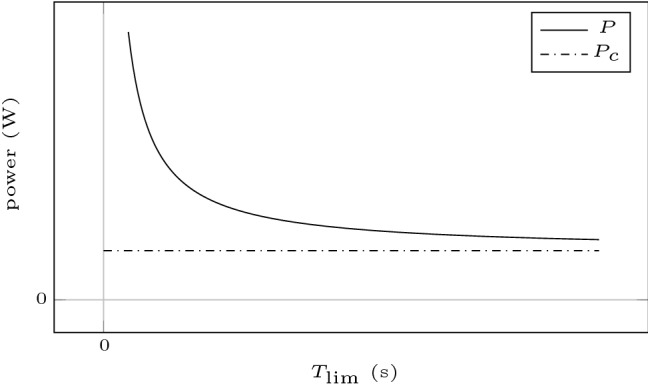
Schematic illustration of the power–endurance relationship of constant power tasks (2). The curvature of this relationship is determined by $W'$ and its asymptote by $P_{\mathrm{c}}$. The power that can be sustained for time $T_\text {lim}$ can be obtained through $P = W'/T_\text {lim} + P_{\mathrm{c}}$

The power–endurance relationship of a constant power task can be described (Monod and Scherrer 1965) by

1 $$\begin{aligned} T_\text {lim} = \frac{W'}{P - P_{\mathrm{c}}} \end{aligned}$$

or equivalently by

2 $$\begin{aligned} P = \frac{W'}{T_\text {lim}} + P_{\mathrm{c}}, \end{aligned}$$

where $T_\text {lim}$ describes the endurance time of a task conducted at constant power *P*, $W'$ describes the curvature constant, and $P_{\mathrm{c}}$, the pole/asymptote of the function, is called critical power (CP). This relation is illustrated schematically in Fig. 1. CP can be interpreted as the maximum power output at which a metabolic steady state can be obtained (Jones et al. 2019). It constitutes an important fatigue threshold in exercise physiology and can be used to analyze, predict, or optimize performance (Craig et al. 2019). Therefore, a reliable and economical estimation is of benefit for athletes, coaches, and exercise physiologists. Its equivalent for isometric or dynamic muscle contractions is the so-called critical torque (CT), which we examine in this work.

Critical torque and the curvature constant $W'$ are usually estimated from multiple submaximal constant torque tests to task failure spread over several days. Burnley et al. (2012), for example, used five trials to determine critical torque of the knee extensors. For intermittent contractions, CT furthermore depends on the work–rest ratio of the periodic loading scheme (Broxterman et al. 2014; Jones and Vanhatalo 2017), which is commonly quantified by the so-called duty cycle. Thus, several CTs need to be estimated for an exercise to obtain a detailed description of the subject. This increases the experimental effort even further.

To reduce the experimental effort, all-out tests have been suggested. Burnley (2009) examined 5 min of maximum intermittent isometric contractions to determine critical torque of the knee extensors and showed that end-torque of these tests closely approximates CT. All-out tests have also been used for other exercises, e.g., for plantar flexion (Abdalla et al. 2018) or for handgrip exercise (Kellawan and Tschakovsky 2014). However, some authors have reported a possible overestimation of CP by the equivalent 3-min all-out test (Muniz-Pumares et al. 2018), which suggests that all-out tests might not be suitable for all subjects or might need to be adapted individually. Kellawan and Tschakovsky (2014), for example, used intermittent isometric contractions lasting 1 s with 2 s rest for 10 min, as they anticipated a longer time to plateau for their experimental setup.

### Purpose

In this work, we propose a model-based approach to reduce the experimental effort when estimating CTs for sustained or intermittent isometric contractions. We employ a phenomenological model of the time course of maximum voluntary isometric contraction (MVIC) torque (Herold et al. 2018) and compute the highest sustainable torque output by solving an optimization problem. We then show that our results are consistent with the steady states obtained when simulating periodic maximum loading schemes. These simulations correspond to all-out tests, which are used to estimate CTs in practice (Burnley 2009).

The estimation of CTs can then be formulated mathematically as a parameter estimation problem, for which the necessary measurements can be obtained in a single testing session compared to several testing sessions when using the traditional approach or an individual adjustment when using all-out tests. As these measurements are subject to random measurement errors, the resulting parameter estimates are random variables (Bock et al. 2013, overview in English; Bock 1987, original work in German). Their statistical properties depend on the experimental setup and may propagate through the model. To reduce the statistical uncertainty of the parameter estimates and consequently of the estimated critical torques, we compute optimized testing sessions. This reduces the experimental effort even further.

## Materials and methods

In this section, we describe the model, the optimization problems, the simulation scenarios (see Table 1), and the optimum experimental design (OED) problems. For readers with a focus away from mathematical modeling, simulation, and optimization, we first provide a textual summary and then invite them to directly proceed to the results section if desired.

### Textual summary

**Fig. 2:**
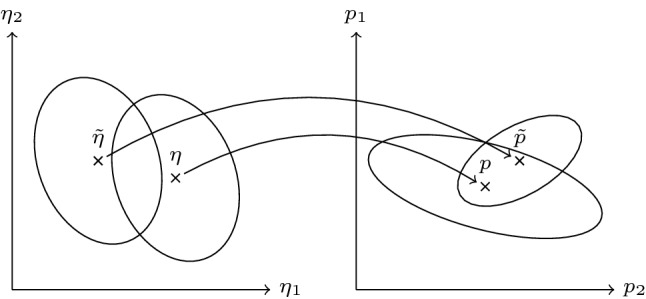
Different experimental conditions result in different parameter estimates and confidence regions thereof. The measurement data obtained by two different experiments are denoted by $\eta$ and ${{\tilde{\eta }}}$. The corresponding parameter estimates are denoted by *p* and ${{\tilde{p}}}$. Confidence regions are illustrated by ellipses. Smaller confidence regions increase the probability of the estimates being close to the ’true’ but unknown value. Redrawn in modified form from Walter (2012)

Previous work (Herold et al. 2018) allows us to predict how MVIC torque of a muscle group decreases and recovers under isometric loading (Eq. (3)). This enables us to find the maximum contraction intensity for which MVIC torque can reach a steady state during intermittent contractions of any desired duty cycle (Eq. (4)), which corresponds to critical torque. However, the mathematical model first needs to be calibrated to the subject’s muscle group (Eq. (7)). For this purpose, measurements of MVIC torque during and after fatiguing contractions have to be taken. These measurements contain varying amount of information depending on the loading scheme and the measurement times (see also Fig. 2). This can be evaluated mathematically before the experiments are conducted (Eq. (8)), which allows us to optimize the experiments with respect to a reliable model calibration (Eq. (15)).

**Table 1:** Overview of simulation scenarios used in this work

Scenario	Explanation
IC	Intermittent contractions lasting 3 s with 2 s rest
ICmax	Intermittent MVIC efforts lasting 3 s with 2 s rest
IC80	Intermittent contractions at 80% of critical torque lasting 3 s with 2 s rest
IC120	Intermittent contractions at 120% of critical torque lasting 3 s with 2 s rest
ITS	Intuitive testing session consisting of a 3 min MVIC effort followed by 2 s MVIC efforts at 10 s, 30 s, 1 min, 2 min, 5 min, 10 min, 15 min, 20 min, 25 min, and 30 min after cessation of the sustained MVIC effort to check the time course of recovery
OTS200	Optimized testing session lasting 1982 s in total with 200 s time under tension and a maximum of 11 contractions
OTS400	Optimized testing session lasting 1982 s in total with 400 s time under tension and a maximum of 11 contractions
RTS	Resistance training session consisting of 5 sets of 5 MVIC efforts lasting 5 s with 5 s inter-repetition rest and 120 s inter-set rest
SC	Sustained contraction
SCmax	Sustained MVIC effort

### Model

For our numerical experiments, we use a phenomenological model of the time course of maximum voluntary isometric contraction torque. We state the ordinary differential equation system and give a short explanation of the components. For a detailed description of the model, we refer to the original paper (Herold et al. 2018).

The model describes the current MVIC torque capacity

3a $$\begin{aligned} h_{{\text {MVIC}}}: [0, T] \rightarrow [0, 1] \end{aligned}$$

of a muscle (or muscle group) at joint level under an external isometric load

3b $$\begin{aligned} u_{{\text {abs}}}: [0, T] \rightarrow [0, 1] \end{aligned}$$

on the time horizon [0, *T*]. MVIC torque capacity and external load are normalized to baseline MVIC torque and are thus dimensionless. Moreover, the ranges of functions specified in this description are restricted to physiological meaningful values. The defining equations of the model are given as

3c $$\begin{aligned} \frac{\text {d}}{\text {d}t}{x}_{{\text {slow}}}(t)&= p_1 (1 - x_{{\text {slow}}}(t)) - p_2 u_{{\text {abs}}}(t) \end{aligned}$$

3d $$\begin{aligned} \frac{\text {d}}{\text {d}t}{x}_{{\text {fast}}}(t)&= p_3 (1 - u_{{\text {abs}}}(t))^{p_4} (1 - x_{{\text {fast}}}(t)) \nonumber \\&\quad -\, p_5 u_{{\text {abs}}}(t) \end{aligned}$$

3e $$\begin{aligned}&h_{{\text {MVIC}}}(t) = x_{{\text {slow}}}(t) x_{{\text {fast}}}(t), \end{aligned}$$

where

3f $$\begin{aligned} x: [0, T] \rightarrow [0, 1]^2 \end{aligned}$$

consists of two dimensionless state variables $x_{\text {fast}}$ and $x_{\text {slow}}$. The model furthermore contains five dimensionless parameters $p_i \in [0, \infty )$ for $i \in \{1, \dots , 5\}$ describing fatigue and recovery properties. Equations (3c) and (3d) are abbreviated by

3g $$\begin{aligned} \frac{\text {d}}{\text {d}t}{x}(t) = f(t, x, p, u_{\text {abs}}) \end{aligned}$$

with the right-hand side $f: [0, T] \times [0, 1]^2 \times [0, \infty )^{5} \times [0, 1] \rightarrow {\mathbb {R}}^2$ in the following. The initial conditions for the states are given by

3h $$\begin{aligned} x(0) = x_0 \in [0, 1]^2. \end{aligned}$$

For an unfatigued muscle, one chooses $x_0 = (1, 1)^\top$. To simulate MVIC efforts, it is favorable to substitute

3i $$\begin{aligned} u_{{\text {abs}}}(t) = u_{{\text {rel}}}(t) h_{{\text {MVIC}}}(t) \end{aligned}$$

and use

3j $$\begin{aligned} u_{{\text {rel}}}: [0, T] \rightarrow [0, 1], \end{aligned}$$

the load relative to the current torque capacity, as input.

The model was validated with a comprehensive set of data from the elbow flexors (Herold et al. 2018). We use the corresponding parameter estimates in this work.

### Model-based estimation of critical torques

We compute the highest sustainable torque output of the elbow flexors by solving the nonlinear program

4a $$\begin{aligned}&\max _{\begin{array}{c} u_{\text {abs}}, x_{\text {slow}}, x_{\text {fast}} \end{array}} u_{\text {abs}}\end{aligned}$$

4b $$\begin{aligned}&\mathrm {s.t.} \ 0 = p_1 (1 - x_{\text {slow}}) - p_2 u_{\text {abs}} \end{aligned}$$

4c $$\begin{aligned}&0 = p_3 (1 - u_{\text {abs}})^{p_4} (1 - x_{\text {fast}}) - p_5 u_{\text {abs}} \end{aligned}$$

4d $$\begin{aligned}&u_{\text {abs}}\le x_{\text {slow}}x_{\text {fast}}= h_{\text {MVIC}} \end{aligned}$$

4e $$\begin{aligned}&0 \le u_{\text {abs}}, x_{\text {slow}}, x_{\text {fast}}\le 1. \end{aligned}$$

Constraints (4b) and (4c) ensure that MVIC torque does not change further and Constraints (4d) and (4e) ensure that the input and the states are feasible.

Exemplarily, we solve the nonlinear program (4) for a sustained contraction (Scenario SC) and for intermittent contractions lasting 3 s with 2 s rest (Scenario IC) as conducted by Burnley (2009) for the knee extensors. For Scenario IC, we use

5a $$\begin{aligned} 0&= 3 (p_1 (1 - x_{\text {slow}}) - p_2 u_{\text {abs}}) + 2 (p_1 (1 - x_{\text {slow}})) \end{aligned}$$

5b $$\begin{aligned} 0&= 3 (p_3 (1 - u_{\text {abs}})^{p_4} (1 - x_{\text {fast}}) - p_5 u_{\text {abs}}) \nonumber \\&\quad +\, 2 (p_3 (1 - x_{\text {fast}})) \end{aligned}$$

instead of Constraints (4b) and (4c). This choice approximates that during one contraction–rest cycle MVIC torque does not change further. The nonlinear program is solved numerically with the sequential least squares programming algorithm by Kraft (1988) provided in SciPy 1.2.1 (Virtanen et al. 2020). To deal with the local maxima provided by the algorithm, we sample the unit cube $[0, 1]^3$ uniformly with 100 grid points in each dimension, use these values as initial guesses to solve the nonlinear program, and choose the solution with the highest objective value.

We verify our results by simulating the model for a sustained MVIC effort (Scenario SCmax) and intermittent MVIC efforts lasting 3 s with 2 s rest (Scenario ICmax) until a plateau of MVIC torque is reached. These simulations correspond to all-out tests proposed by Burnley (2009). Thus, the end-test torques provide estimates of CTs. To terminate our simulations during Scenario SCmax because a plateau is reached, we demand $| \frac{\text {d}}{\text {d}t}{x}_{{\text {slow}}} | \le 10^{-6}$ and $| \frac{\text {d}}{\text {d}t}{x}_{{\text {fast}}} | \le 10^{-6}$. To terminate our simulations during Scenario ICmax because a plateau is reached, we demand that the torque at the beginning of two adjacent contractions does not differ more than $10^{-6}$. These thresholds are low enough to ensure that a steady state has been obtained, but do not require excessive computation times. Afterwards, we compare the computed steady states to the end-test torques of simulated 5-min all-out tests for both scenarios.

To illustrate that the thus determined steady states separate domains of contraction intensity, we recreate the experimental setting of Burnley et al. (2012) for the elbow flexors. We simulate intermittent contractions lasting 3 s with 2 s rest at 80% (Scenario IC80) and at 120% (Scenario IC120) of the previously determined critical torque on a time horizon of 60 min or until MVIC torque drops below target torque.

Finally, to demonstrate the full potential of our approach, we compute the highest sustainable torque output of the elbow flexors for intermittent contractions depending on the duty cycle. The duty cycle is defined as the ratio $t_{\mathrm{c}} / (t_{\mathrm{c}} + t_{\mathrm{r}}),$ where $t_{\mathrm{c}}$ denotes the duration of a contraction and $t_{\mathrm{r}}$ denotes the inter-repetition rest. Therefore, we solve the nonlinear program (4) for 100 duty cycles distributed uniformly in [0, 1] and plot the results.

### Optimized testing sessions

#### Mathematical background

In this work, we use the parameters obtained for the elbow flexors during the model development for illustrative purposes. To use our approach in real life, the model needs to be calibrated to the subject and model parameters *p* have to be provided. Therefore, one conducts experiments and fits the model to measurement data $\eta \in {\mathbb {R}}^{n_m}$.

We assume the model to be correct and the measurement errors $\epsilon _i$ to be additive, independent, and normally distributed with expected value $\mu _i = 0$ and standard deviation $\sigma _i \in (0, \infty )$. Consequently, the $n_m$ measurements $\eta _i$ can be modeled as

6 $$\begin{aligned} \eta _i = h(t_i, x^{*}(t_i), p^*) + \epsilon _i, \qquad i = 1, \dots , n_m, \end{aligned}$$

where $h: [0, T] \times [0, 1]^{2} \times [0, \infty )^{5} \rightarrow [0, 1]$ denotes the measurement function, $t_i$ is the time of the measurement, $p^*$ are the ’true’ but unknown parameter values, and $x^{*}$ are the corresponding states. Following these assumptions, the maximum likelihood estimator of $p^*$ can be obtained by solving the nonlinear weighted least squares problem

7a $$\begin{aligned}&\min _{p, x(\cdot )} \frac{1}{2} \sum _{i=1}^{n_m} \left( \frac{h(t_i, x(t_i), p) - \eta _i}{\sigma _i} \right) ^2 \end{aligned}$$

7b $$\begin{aligned}&\mathrm {s.t.} \ \frac{\text {d}}{\text {d}t}{x}(t) = f(t, x, p, u_{\text {abs}}) \end{aligned}$$

7c $$\begin{aligned}&x(0) = x_0 \in [0, 1]^2. \end{aligned}$$

The loading scheme is described by the control function $u_{\text {abs}}$. This is known during the parameter estimation, as the experiment was already conducted.

Since the measurement data $\eta$ are subject to random measurement errors, the solution ${\hat{p}}$ of problem (7) is a random variable (Bock et al. 2013, overview in English; Bock 1987, original work in German). Under certain regularity assumptions, one can approximate its variance–covariance matrix by

8 $$\begin{aligned} C = (J^\top J)^{-1}\in {\mathbb {R}}^{5 \times 5} \end{aligned}$$

with the Jacobian $J =$

$$\begin{aligned} \begin{pmatrix} \frac{1}{\sigma _1} \frac{\text {d}h}{\text {d}p_1}(t_1, x(t_1), p) &{}\quad \dots &{}\quad \frac{1}{\sigma _1} \frac{\text {d}h}{\text {d}p_{5}}(t_1, x(t_1), p) \\ \vdots &{}\quad \ddots &{}\quad \vdots \\ \frac{1}{\sigma _{n_m }} \frac{\text {d}h}{\text {d}p_1}(t_{n_m}, x(t_{n_m}), p) &{}\quad \dots &{}\quad \frac{1}{\sigma _{n_m}} \frac{\text {d}h}{\text {d}p_{5}}(t_{n_m}, x(t_{n_m}), p) \end{pmatrix} \end{aligned}$$

evaluated at $p = {\hat{p}}$. The approximation *C* describes a confidence ellipsoid around the parameter estimates ${\hat{p}}$. It therefore allows us to analyze the statistical quality of these estimates. For example, the diagonal entries of *C* approximate the variances of the parameter estimates (Pukelsheim 1993).

The variance–covariance matrix *C* depends on the loading scheme described by $u_{\text {abs}}$ and on the current guess ${\hat{p}}$. However, it does not depend on the measurement data $\eta$. This enables us to design experiments which reduce the statistical uncertainty of the estimates. This idea is illustrated in Fig. 2 and realized in the following.

#### Experimental setting

Allen et al. (1995) report a mean empirical coefficient of variation of 3.78% for individual 2–3 s MVIC torque measurements of the elbow flexors in the unfatigued state. The experimental setup was similar to that used for the model development (Herold et al. 2018). To account for the increased torque variability observed during fatigue (Contessa et al. 2009), we use this value as absolute standard deviation of the measurement errors. The measurement error $\varepsilon$ of a 2 s MVIC relative torque measurement is thus assumed to be additive, independent, and identically normally distributed with mean zero and standard deviation $\sigma _{2\text {s}} = 0.0378$. The subscript denotes the duration of the contraction.

State-of-the-art force transducers can provide high-frequency measurement data. Hence, we may assume that measurements of MVIC torque can be obtained continuously if the subject can accurately estimate the applied torque $u_{\text {abs}}$ in relation to its current torque capacity $h_{\text {MVIC}}$, i.e., if the subject can accurately estimate

9 $$\begin{aligned} u_{\text {rel}}(t) = \frac{u_{\text {abs}}(t)}{h_{\text {MVIC}}(t)}. \end{aligned}$$

This yields the measurement function

10 $$\begin{aligned} h(t) = u_{\text {rel}}(t) h_{\text {MVIC}}(t). \end{aligned}$$

However, estimating $u_{\text {rel}}$ for submaximal contractions is a challenging task (Banister 1979), which is additionally influenced by fatigue (Jones and Hunter 1983). Therefore, we increase the standard deviation of measurements taken at submaximal contractions according to

11 $$\begin{aligned} \sigma (t) = \sigma _{2\text {s}} (2 - u_{\text {rel}}(t)). \end{aligned}$$

For maximum contractions ($u_{\text {rel}}= 1$), we again obtain the uncertainty observed by Allen et al. (1995).

To allow a numerical treatment of the continuous measurements (Janka 2015), we choose a sufficiently fine measurement grid $(t_j)_{j \in \{1, \dots , n_m\}}$ and approximate the corresponding discretized standard deviations by

12 $$\begin{aligned} \sigma _j = \sigma _{2\text {s}} (2 - u_{\text {rel}}(t_j)) \sqrt{\frac{2}{\varDelta t_j}}, \quad j \in \{1, \dots , n_m\}. \end{aligned}$$

Here, $\varDelta t_j$ denotes the duration of the measurement following the grid point $t_j$. The weighting $\sqrt{\frac{2}{\varDelta t_j}}$ is necessary to take into account the coarseness of the measurement grid. For example, doubling $\varDelta t_j$ would then correspond to weighting the measurement taken at $t_j$ twice. This furthermore allows us to treat measurements of any duration.

Moreover, we introduce a measure of the total time under tension (TUT)

13 $$\begin{aligned} I_{\text {TUT}}(t) = \int _0^{t} {\left\{ \begin{array}{ll} 0 &{}\quad \text {if } u_{\text {rel}}(\tau ) = 0 \\ 1 &{}\quad \text {else} \\ \end{array}\right. } \text {d}\tau \end{aligned}$$

to allow a fair comparison between different loading schemes. If we verify $u_{\text {rel}}\in \{0, 1\}$ for our solutions, we can use

14 $$\begin{aligned} I_{\text {TUT}}(t) = \int _0^{t} u_{\text {rel}}(\tau ) \text {d}\tau \end{aligned}$$

as an equivalent measure.

#### Mathematical problem formulation

We use a multi-stage formulation on $n_s \ge 2$ stages—denoted by superscripts $i \in \{1, \dots , n_s\}$—to model the loading schemes (Herold et al. 2018). We define

15a $$\begin{aligned} J = (J^1, \dots , J^{n_s})^\top , \end{aligned}$$

with

15b $$\begin{aligned} J^i_j = \frac{1}{\sigma ^i_j} \left. \left( \frac{\partial h^i}{\partial x^i} x^i_p(t^i_j) + \frac{\partial h^i}{\partial p}(t^i_j) \right) \right| _{p = {\hat{p}}}, \end{aligned}$$

where the sensitivities of the model states w.r.t. the parameters are denoted by $x^i_p(t) = \frac{\text {d}x^i}{\text {d}p}(t)$ and the measurement times on stage *i* are denoted by $t_j^i$. To track TUT, we introduce an additional state $I_\text {TUT}$ defined as in Eq. 13. The multi-stage OED problem can then be formulated as

15c $$\begin{aligned}&\min _{\begin{array}{c} x^i(\cdot ), x_p^i(\cdot ), I_{\text {TUT}}^i(\cdot )\\ u^i_{\text {abs}}(\cdot ), T^i \end{array}} \frac{1}{5} \mathrm {tr}((J^\top J)^{-1}) \end{aligned}$$

15d $$\begin{aligned}&\text {s.t.} \ 0.01 \text { s} \le T^{i} \text { for } i \in \{1, \dots , n_s\} \end{aligned}$$

15e $$\begin{aligned}&\sum _{i = 1}^{n_s} T^{i} \le C_T \end{aligned}$$

15f $$\begin{aligned}&I^{n_s}_{\text {TUT}}(T^{n_s}) \le C_\text {TUT} \end{aligned}$$

15g $$\begin{aligned}&x^0(0) = (1, 1)^\top \end{aligned}$$

15h $$\begin{aligned}&x_p^0(0) = (0, 0)^\top \end{aligned}$$

15i $$\begin{aligned}&I^{0}_{\text {TUT}}(0) = 0 \end{aligned}$$

15j $$\begin{aligned}&\text {and for} \ i \in \{2, \dots , n_s\}: \nonumber \\&x^i(0) = x^{i - 1}(T^{i - 1}) \end{aligned}$$

15k $$\begin{aligned}&x_p^i(0) = x_p^{i - 1}(T^{i - 1}) \end{aligned}$$

15l $$\begin{aligned}&I^{i}_{\text {TUT}}(0) = I^{i - 1}_{\text {TUT}}(T^{i - 1}) \end{aligned}$$

15m $$\begin{aligned}&\text {and} \ i \in \{1, 3, \dots , n_s - 2, n_s\} \text { and } t \in [0, T^{i}]: \nonumber \\&\frac{\text {d}}{\text {d}t}x^i(t) = f(t, x^i(t), p, u^i_{\text {abs}}(t)) \end{aligned}$$

15n $$\begin{aligned}&\frac{\text {d}}{\text {d}t}x^i_p(t) = \frac{\partial f}{\partial x^i} x_p^i(t) + \frac{\partial f}{\partial p} \end{aligned}$$

15o $$\begin{aligned}&\frac{\text {d}}{\text {d}t}I^{i}_{\text {TUT}}(t) = \frac{u^i_{\text {abs}}(t)}{h^i_{\text {MVIC}}(t)} \end{aligned}$$

15p $$\begin{aligned}&0 \le u^i_{\text {abs}}(t) \le h_{\text {MVIC}}^i(t) \end{aligned}$$

15q $$\begin{aligned}&\text {and for} \ i \in \{2, 4, \dots , n_s - 3, n_s - 1\} \text { and } t \in [0, T^{i}]: \nonumber \\&\frac{\text {d}}{\text {d}t}x^i(t) = f(t, x^i(t), p, 0) \end{aligned}$$

15r $$\begin{aligned}&\frac{\text {d}}{\text {d}t}x^i_p(t) = \frac{\partial f}{\partial x^i} x_p^i(t) + \frac{\partial f}{\partial p} \end{aligned}$$

15s $$\begin{aligned}&\frac{\text {d}}{\text {d}t}I^{i}_{\text {TUT}}(t) = 0, \end{aligned}$$

with $C_T$ being the upper bound on the sum of all stage durations $T^i$ and $C_\text {TUT}$ being the upper bound on the sum of all stage-wise TUTs. The lower bounds on the stage durations $T^i$ (15d) are necessary to avoid a division by zero in Eq. (12), as the stage durations are being optimized. Minimizing the trace of $(J^\top J)^{-1}$ corresponds to minimizing the average variance of the parameter estimates (Pukelsheim 1993). To weight the parameters equally, we scale all parameters to 1 beforehand. Furthermore, the input function $u_{\text {abs}}$ is replaced by $u_{\text {rel}}$ according to Eq. (3i) to allow a straightforward implementation of the experimental setting presented above. Table 2 gives an overview of the symbols used in the problem formulation.

**Table 2:** Overview of symbols used in OED problem (15)

Symbol	Interpretation
$C_T$	Upper bound on total time
$C_\text {TUT}$	Upper bound on total TUT
*f*	Right-hand side of ODE system
$h^i$	Measurement function
$h^i_{\text {MVIC}}$	MVIC torque
*i*	Stage index
$I^i_\text {TUT}$	Time under tension
$J^i$	Jacobian
$n_s$	Number of stages
*p*	Parameters
${\hat{p}}$	Current parameter guess
$\sigma ^i_j$	Standard deviation of measurement error
*t*	Time
$T^i$	Stage duration
$t^i_j$	Measurement time
$\text {tr}$	Trace of matrix
$u^i_{\text {abs}}$	External torque
$x^i$	State variables
$x^i_p$	Sensitivities of states

To solve the problem, we employ a first-discretize-then-optimize strategy. We use a direct single shooting approach to reduce the problem to a finite-dimensional form and employ DAESOL (Bauer et al. 1997, overview in English; Bauer 1999, detailed description in German) for integration of the ordinary differential equation system and sensitivity generation via internal numerical differentiation (Bock 1981). The resulting nonlinear program is then solved with the sequential quadratic programming method SNOPT (Gill et al. 2005). We use the software package VPLAN (Bock et al. 2013, overview in English; Körkel 2002, detailed description in German) to carry out all steps.

#### Numerical experiments

To illustrate the benefits of OED, we compare the uncertainties of the parameters resulting from an intuitive testing session (Scenario ITS) with those resulting from algorithmically designed optimized testing sessions (Scenarios OTS200 and OTS400). Scenario ITS consists of a 3 min MVIC effort followed by 2 s MVIC efforts at 10 s, 30 s, 1 min, 2 min, 5 min, 10 min, 15 min, 20 min, 25 min, and 30 min after cessation of the sustained MVIC effort to check the time course of recovery. Thus, it lasts 1982 s in total, of which 200 s are TUT. This loading scheme is motivated by comparable sessions conducted to examine fatigue and recovery of skeletal muscle (Gandevia et al. 1996; Søgaard et al. 2006).

The optimized sessions are computed as described above with Scenario ITS as initial guess. For Scenario OTS200, to allow a fair comparison, we limit the maximum number of contractions to 11 (which implies $n_s = 21$), the total time to $C_T =$ 1982 s, and the time under tension to $C_\text {TUT} =$ 200 s as in the intuitive testing session. On each odd numbered model stage, we use constant controls and employ $n_m^i = 100$ discrete measurements to approximate the continuous measurements of the force transducer. Even numbered stages are considered rest periods. For Scenario OTS400, we use the same setup but increase the limit of the time under tension to $C_\text {TUT} =$ 400 s.

Additionally, we demonstrate how the uncertainties of the parameters propagate through the model. As no measurement data are available, we assume that the intuitive and the optimized testing sessions resulted in the same parameter estimates *p* but with different standard deviations $\sigma _p$. We then draw 10,000 random samples from ${\mathcal {N}}(p, \sigma _p^2)$ and simulate two different scenarios with these perturbed parameters. We redraw realizations with negative parameters, since the model is not evaluable for those. The probability for which is 0.5% when using the standard deviations resulting from the intuitive testing session and 0% when using those resulting from the optimized testing sessions. First, we simulate Scenario ICmax for 60 min, as we have done to estimate critical torque. Second, we simulate a possible resistance training session consisting of 5 sets of 5 maximum contractions lasting 5 s with 5 s inter-repetition rest and 120 s inter-set rest (Scenario RTS). For both scenarios, we then analyze the differences of end-MVIC torque of the perturbed settings to the nominal setting *p*. The kernel density estimates used for this analysis were obtained using the gaussian_kde function of SciPy 1.2.1 (Virtanen et al. 2020) with Scott’s rule of thumb for bandwidth selection.

## Results

In the following, we provide the results of our computations. For readers who skipped the methods section, we redescribe our approach without the mathematical details. We refer to Table 1 for a concise overview of the simulation scenarios.

### Model-based estimation of critical torques

We compute the highest sustainable torque output of the elbow flexors by solving the optimization problem (4) for a sustained contraction (Scenario SC) and for intermittent contractions lasting 3 s with 2 s rest (Scenario IC) as conducted by Burnley (2009) for the knee extensors. For Scenario SC, the solution is 27.99% of baseline MVIC torque. For Scenario IC, the solution is 41.01% of baseline MVIC torque.

**Fig. 3:**
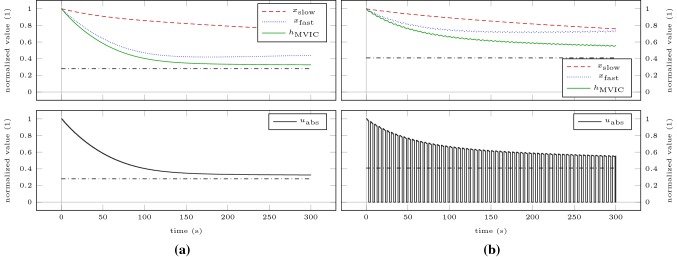
Model response obtained by simulating scenarios SCmax (**a**) and ICmax (**b**) for 5 min. The bottom row illustrates the absolute torque input as predicted by the model. The dash-dotted lines represent the steady states obtained by simulating the scenarios until a plateau of MVIC torque is reached

We verify our results by simulating the model for a sustained MVIC effort (Scenario SCmax) and intermittent MVIC efforts lasting 3 s with 2 s rest (Scenario ICmax) until a plateau of MVIC torque is reached. These simulations correspond to all-out tests proposed by Burnley (2009). Thus, the end-test torques provide estimates of CTs. Figure 3 shows the model response obtained by simulating Scenarios SCmax and ICmax for 5 min. For both scenarios, a steady state according to our definition in “Model-based estimation of critical torques” is not obtained after 5 min. End-test torques are 32.59% of baseline MVIC torque for the sustained contraction and 54.72% of baseline MVIC torque for the intermittent contractions. Simulating the scenarios on a time horizon of 60 min results in steady states. The sustained contraction levels off at 28.01% of baseline MVIC torque. The intermittent contractions level off at 40.89% of baseline MVIC torque. Both solutions are similar to the results obtained by solving the optimization problem (4). To illustrate the discrepancy to the end-test torques obtained by the 5-min all-out test, these steady states are depicted as dash-dotted lines in Fig. 3.

**Fig. 4:**
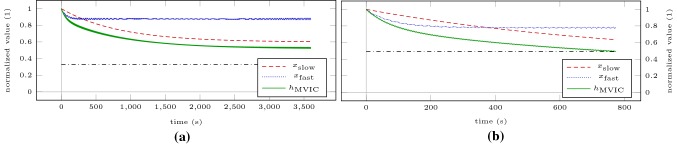
Model response obtained by simulating Scenario IC80 for 60 min (**a**) and Scenario IC120 until MVIC torque drops below target torque (**b**). The dash-dotted lines represent the target torques of the intermittent contractions. The torque inputs have been omitted for these plots as due to the high number of intermittent contractions the plots would become illegible

To illustrate that the thus determined steady states separate domains of contraction intensity, we recreate the experimental setting of Burnley et al. (2012) for the elbow flexors. We simulate intermittent contractions lasting 3 s with 2 s rest at 80% (Scenario IC80) and at 120% (Scenario IC120) of the previously determined steady states on a time horizon of 60 min or until MVIC torque drops below target torque. These torques correspond to 32.71% and 49.07% of baseline MVIC torque. Figure 4 shows the model response obtained by simulating both scenarios. During Scenario IC80, MVIC torque approaches a steady state above the target torque at 52.60% of baseline MVIC torque. During Scenario IC120, MVIC torque falls below target torque at $t =$ 773 s.

**Fig. 5:**
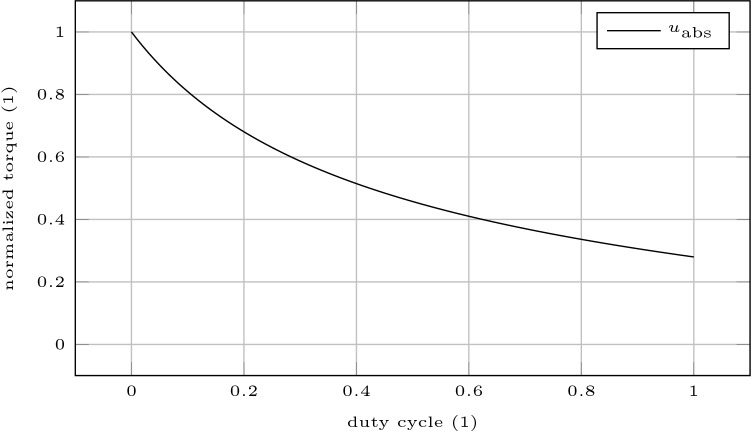
Highest sustainable torque output of the elbow flexors for intermittent contractions depending on the duty cycle. The duty cycle denotes the ratio $t_{\mathrm{c}} / (t_{\mathrm{c}} + t_{\mathrm{r}}),$ where $t_{\mathrm{c}}$ denotes the duration of a contraction and $t_{\mathrm{r}}$ denotes the inter-repetition rest

Finally, to demonstrate the full potential of our approach, we compute the highest sustainable torque output of the elbow flexors for intermittent contractions depending on the duty cycle. The duty cycle is defined as the ratio $t_{\mathrm{c}} / (t_{\mathrm{c}} + t_{\mathrm{r}}),$ where $t_{\mathrm{c}}$ denotes the duration of a contraction and $t_{\mathrm{r}}$ denotes the inter-repetition rest. Figure 5 depicts this dependency.

### Optimized testing sessions

In this work, we use the parameters obtained for the elbow flexors during the model development for illustrative purposes. To use our approach in real life, the model needs to be calibrated to the subject and model parameters have to be provided. Therefore, one conducts experiments and fits the model to measurement data. The statistical properties of the parameter estimates depend on the experimental setup but not on the measurement data. Thus, we can design experiments which reduce the statistical uncertainty of the estimates.

To illustrate the benefits of optimum experimental design (OED), we compare the uncertainties of the parameters resulting from an intuitive testing session (Scenario ITS) with those resulting from algorithmically designed optimized testing sessions (Scenarios OTS200 and OTS400). The intuitive session consists of a 3 min MVIC effort followed by 2 s MVIC efforts at 10 s, 30 s, 1 min, 2 min, 5 min, 10 min, 15 min, 20 min, 25 min, and 30 min after cessation of the sustained MVIC effort to check the time course of recovery. Thus, it lasts 1982 s in total, of which 200 s are time under tension (TUT). This scenario is motivated by comparable sessions conducted to examine fatigue and recovery of skeletal muscle (Gandevia et al. 1996; Søgaard et al. 2006). The optimized sessions are computed as described in “Optimized testing sessions”. For Scenario OTS200, to allow a fair comparison, we limit the maximum number of contractions to 11, the total time to 1982 s, and TUT to 200 s. For Scenario OTS400, we increase the limit of TUT to 400 s.

**Fig. 6:**
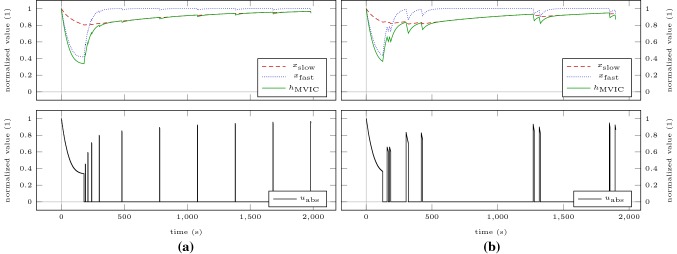
Model response obtained by simulating the intuitive testing session ITS (**a**) and the optimized testing session OTS200 (**b**). The bottom row illustrates the absolute torque input as predicted by the model. All contractions are maximal

**Fig. 7:**
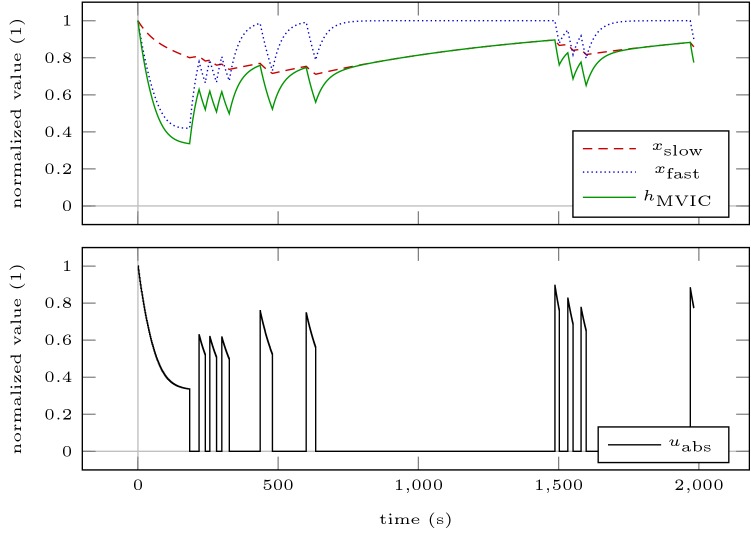
Model response obtained by simulating the optimized testing session OTS400. The bottom row illustrates the absolute torque input as predicted by the model. All contractions are maximal

**Fig. 8:**
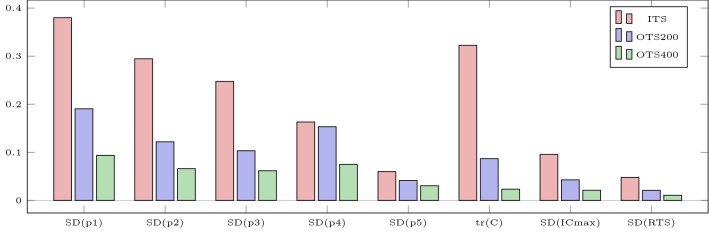
Estimated standard deviations of the model parameters SD(p) and trace $\mathrm {tr}(C)$ of the variance–covariance matrix resulting from the intuitive (Scenario ITS) and the optimized testing sessions (Scenarios OTS200 and OTS400). All parameters were scaled to 1 for the OED computations. Furthermore, SD(ICmax) and SD(RTS) denote the standard deviations of the differences of end-MVIC torque of the 10,000 perturbed settings from the nominal setting *p* using the parameter uncertainties resulting from the intuitive testing session (ITS) and from the optimized testing sessions (OTS200 and OTS400)

Figures 6 and 7 illustrate the loading schemes and the model response of the intuitive and the optimized testing sessions. Figure 8 illustrates the estimated standard deviations of the model parameters and the trace of the variance–covariance matrix resulting from these sessions.

Additionally, we demonstrate how the uncertainties of the parameters propagate through the model. As no measurement data is available, we assume that the intuitive and the optimized testing sessions resulted in the same parameter estimates *p* but with different standard deviations $\sigma _p$. We then draw 10,000 random samples from the corresponding  normal distribution and simulate two different scenarios with these perturbed parameters.

**Fig. 9:**
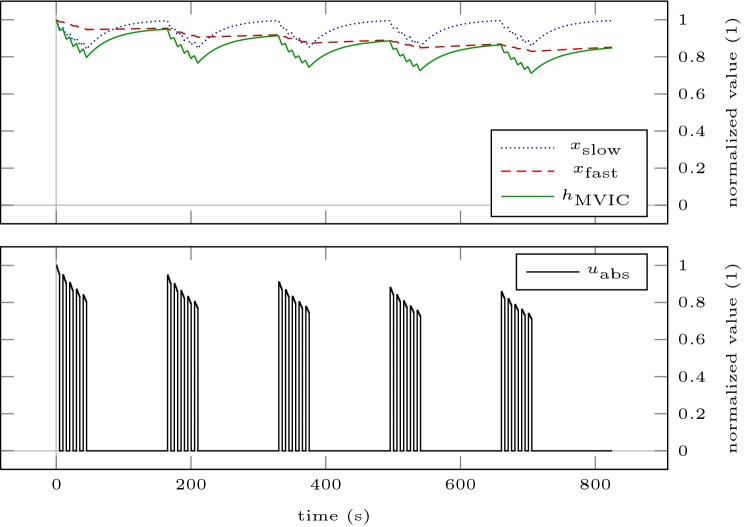
Model response obtained by simulating Scenario RTS. The bottom row illustrates the absolute torque input as predicted by the model. This is one of two scenarios used to examine how the parameter uncertainties propagate through the model

**Fig. 10:**
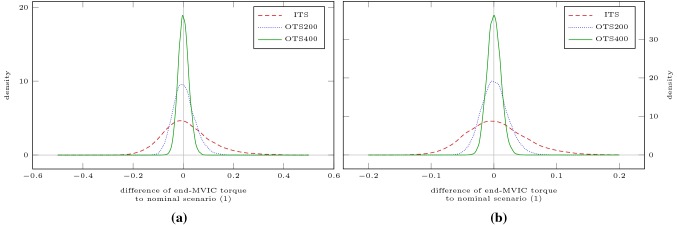
Kernel density estimates obtained by analyzing the differences of end-MVIC torque of the 10,000 perturbed settings from the nominal setting *p* using the parameter uncertainties resulting from the intuitive testing session (ITS) and from the optimized testing sessions (OTS200 and OTS400). The left plot (**a**) shows the results for Scenario ICmax. The right plot (**b**) shows the results for Scenario RTS. The parameter uncertainties of the optimized testing sessions result in sharper peaks around the mean value 0

First, we simulate Scenario ICmax for 60 min, as we have done to estimate critical torque. Second, we simulate a possible resistance training session consisting of 5 sets of 5 maximum contractions lasting 5 s with 5 s inter-repetition rest and 120 s inter-set rest (Scenario RTS). This loading scheme is illustrated in Fig. 9. Figure 10 shows the kernel density estimates of the differences of end-MVIC torque of the perturbed settings to the nominal setting *p* for both scenarios. Figure 8 illustrates the standard deviations of those differences.

## Discussion

### Model-based estimation of critical torques

Our results show that our approach yields similar estimates for CTs as the all-out tests proposed by Burnley (2009), if those are conducted for a sufficiently long duration. On the one hand, this verifies our model-based approach from a practical point of view. On the other hand, this augments the theoretical justification of the all-out tests, as the optimization problem (4) arises purely from the definition of CT as the highest sustainable torque output.

Using the experimental setup of Burnley et al. (2012), we can illustrate that the determined steady states indeed separate domains of contraction intensity. This is remarkable, as the concept of critical torque was not specifically implemented into the model (Herold et al. 2018) but rather emerges naturally. These results underline the importance of critical torque as an important fatigue threshold in exercise physiology.

Our results also show that the durations needed to actually attain a steady state for the elbow flexors are longer than the 5 min proposed by Burnley (2009) for the knee extensors. Yet, durations of 60 min can not be used in real experiments. Therefore, to a certain degree, an overestimation of critical torque is probable when using all-out tests. This is in line with other authors who found that the equivalent 3-min all-out tests might overestimate critical power (Muniz-Pumares et al. 2018). In our simulations, this overestimation is more pronounced for the intermittent contractions than for the sustained contraction. Thus, we propose that the durations of all-out tests are adjusted to the subject and the exercise if conventional methods for the estimation of critical torque are used. However, we emphasize that these adjustments are not necessary when using our model-based approach.

Previous studies have shown the knee extensors to be more fatigable than the elbow flexors. For examples, we refer to Vernillo et al. (2017) for a comparison of MVIC efforts and to Frey-Law and Avin (2010) for an analysis of endurance times. Our results are consistent with these findings, as the estimated critical torques of the elbow flexors are higher than the corresponding ones for the knee extensors. Burnley et al. (2012), for example, report a mean critical torque of the knee extensors of 34% of baseline MVIC torque for intermittent isometric contractions lasting 3 s with 2 s rest. For the same contraction scheme, our computations yield a critical torque of 41% of baseline MVIC torque for the elbow flexors. Moreover, Hendrix et al. (2009a) give a mean critical torque of 17.6% of baseline MVIC torque for sustained contractions of the knee extensors. For the same contraction scheme, our computations yield a critical torque of 28% of baseline MVIC torque for the elbow flexors.

Different experimental conditions (e.g., joint angles) complicate a straightforward comparison of our results to other studies examining critical torques of the elbow flexors. Furthermore, deducing a clear trend from the considered studies proves to be challenging. Hendrix et al. (2009b), for instance, report a mean critical torque of 17.6% of baseline MVIC torque for sustained contractions of the elbow flexors, compared to a mean value of 26.3% by Hendrix et al. (2010). In contrast, for a continuous isometric contraction of the elbow flexors that can be sustained for 60 min, Hagberg (1981) gives a mean contraction intensity of only 8.2% of baseline MVIC torque and Sato et al. (1984) give only 10.3% of baseline MVIC torque. For intermittent isometric contractions lasting 2 s with 2 s rest that can be sustained for 60 min, Hagberg (1981) reports a mean value of 25.1% of baseline MVIC torque. The high variability of reported values points out the need for further research, as it is unclear whether those result from inter-individual or from methodological differences.

### Optimized testing sessions

Figure 8 illustrates that the optimized testing session OTS200 decreases the uncertainties of all parameters substantially compared to the intuitive session ITS. Scenario OTS200 consists of a prolonged MVIC effort in the beginning and 8 MVIC efforts afterwards. Those are of slightly longer duration and distributed differently than in the intuitive testing session. However, the constraints imposed on the total time and on the time under tension seem to be too restrictive to allow an actual identification of the parameters.

Therefore, we increase the limit on TUT for the OED problem OTS400. Here, all parameters can be identified with reasonable accuracy (SD $\le$ 10%). We acknowledge that a testing session lasting more than 30 min with almost 7 min of maximum contractions is taxing on the subjects. Nevertheless, we are certain that the benefit of determining several CTs in a single session outweighs this aspect. In case a testing session has to be stopped, the measurement data does not have to be discarded but can be used in a multi-experiment setting (Schlöder and Bock 1983) for subsequent parameter estimations. This is a further advantage of our approach.

These scenarios serve as representative real-world examples and the improvements in Fig. 8 demonstrate what an algorithmic design of experiments is capable of. Depending on the preferences of the experimenters and the subjects, further experiments could be designed in a straightforward manner reducing the experimental effort considerably.

As the parameters of the model do not bear a direct physiological meaning, designing specific experiments to reduce their uncertainties might seem unnecessary to practitioners. It is important to keep in mind that the uncertainties of the parameter estimates propagate through the model and influence other quantities of interest. Our simulations of the perturbed settings illustrate this influence on the estimate of critical torque and end-MVIC torque of a potential resistance training session. Scenario RTS was chosen as a further illustrative example since the model was designed to optimize such scenarios (Herold et al. 2018). The standard deviations in Fig. 8 and the kernel density estimates of Fig. 10 demonstrate clearly how reduced parameter uncertainties improve the model prediction.

## Limitations and future work

Our approach is not free of limitations and several directions of future research are possible.

First, we do not formally prove that the solutions of the optimization problem (4) are approached and obtained during an all-out test for all periodic loading schemes and parameter values, as this is beyond the scope of this work. Rather this has to be ensured individually, as we did for the two scenarios examined here.

Second, we can not provide an estimate of the curvature parameter $W'$. The intuitive connection to impulse above end-test torque could not be verified by Burnley (2009). Thus, at the moment, if an estimate of $W'$ is desired, conventional submaximal constant power tests to failure have to be employed.

Third, due to the phenomenological nature of the model it does not provide insight into the metabolic or systemic profile of the subject and the tested muscles. Therefore, it remains to be examined experimentally which mechanisms are responsible for the model calibrated to the elbow flexors reaching its steady state later than the knee extensors examined by Burnley (2009).

Fourth, the model is currently limited to isometric contractions only (Herold et al. 2018). It would be interesting to see if a similar steady state behavior emerges naturally after the model has been extended to isokinetic contractions or contractions with dynamic constant external resistance. Thus, incorporating a velocity dependency into the model could save additional experimental effort when dealing with these contractions. Morel et al. (2019), for example, showed that the asymptote of MVIC torque during an isokinetic all-out test depends on the contraction velocity. Intriguingly, during all-out tests, the time course of power bears strong resemblance with the time course of torque [see, for example, Fig. 1 in Vanhatalo et al. (2007)]. This might indicate a possible application of the model in power-measured exercises. Eriksson et al. (2016) already demonstrated that a model-based approach is also feasible for whole-body exercise. The authors developed a mathematical model of fatigue during whole-body exercise and qualitatively showed that their model can be used to determine critical power.

Last, we only consider unfatigued muscles. Yet, it is also possible to use our approach for prefatigued muscles by treating the initial values $x_0$ as additional parameters during the parameter estimation. Then, studies investigating the influence of fatigue on CT similar to those of Vanhatalo and Jones (2009) or Clark et al. (2018) are possible. This might provide further understanding and quantification of the interaction of fatigue and fatigability.

## Conclusion

We are able to estimate CTs for sustained and intermittent isometric contractions with a model-based approach in a single optimized testing session. This reduces the experimental effort considerably compared to conventional testing.

## Abbreviations

CP: Critical power
CT: Critical torque
MVIC: Maximum voluntary isometric contraction
OED: Optimum experimental design
SD: Standard deviation
TUT: Time under tension
